# Ten-year trends of the clinicopathological characteristics, surgical treatments and survival outcomes of operable lung cancer patients in monocenter: a retrospective cohort study

**DOI:** 10.3389/fmed.2023.1133344

**Published:** 2023-04-26

**Authors:** Dechang Zhao, Xiaotian He, Rusi Zhang, Zirui Huang, Yingsheng Wen, Xuewen Zhang, Gongming Wang, Guangran Guo, Lianjuan Chen, Lanjun Zhang

**Affiliations:** ^1^State Key Laboratory of Oncology in South China, Collaborative Innovation Center for Cancer Medicine, Guangzhou, China; ^2^Department of Thoracic Surgery, Sun Yat-sen University Cancer Center, Guangzhou, China; ^3^Department of Anesthesiology, Sun Yat-sen University Cancer Center, Guangzhou, China

**Keywords:** lung cancer, trends, clinicopathological characteristics, surgical treatments, survival outcomes

## Abstract

**Background:**

Lung cancer is one of the cancers with the highest morbidity and mortality. During the last decade, the trends of clinical characteristics, surgical treatments and survival of lung cancer patients in China have remained unclear.

**Methods:**

All lung cancer patients operated on from 2011 to 2020 were identified in a prospectively maintained database of Sun Yat-sen University Cancer Center.

**Results:**

A total of 7,800 lung cancer patients were included in this study. Within the past 10 years, the average age at diagnosis of the patients remained stable, the proportion of asymptomatic, female and nonsmoking patients increased, and the average tumor size decreased from 3.766 to 2.300 cm. In addition, the proportion of early stage and adenocarcinoma increased, while that of squamous cell carcinoma decreased. Among the patients, the proportion of patients having video-assisted thoracic surgery increased. More than 80% of the patients underwent lobectomy and systematic nodal dissection over the 10 years. Additionally, both the average postoperative length of stay and 1-, 3-, and 6-month postoperative mortality decreased. Moreover, the 1-, 3-, and 5-year overall survival (OS) rates of all the operable patients increased from 89.8, 73.9, and 63.8% to 99.6, 90.7, and 80.8%, respectively. The 5-year OS rates of the patients with stage I, II, and III lung cancer were 87.6, 79.9, and 59.9%, respectively, which were higher than those in other published data.

**Conclusion:**

There were significant changes in the clinicopathological characteristics, surgical treatments and survival outcomes of the patients with operable lung cancer from 2011 to 2020.

## Introduction

According to Global Cancer Statistics 2020, lung cancer is the second most commonly diagnosed cancer and the leading cause of cancer death (1). In China, lung cancer still accounts for the highest cancer mortality and morbidity. Additionally, the mortality and morbidity of lung cancer are expected to increase over the next 20 years, imposing a heavy burden on China (2). In most countries, the 5-year survival of lung cancer was in the range of 10–19% during 2010–2014, and the survival trends between 1995–1999 and 2000–2014 remained largely unchanged. However, in China, survival has increased by more than 10%, and it previously was 19.8% during 2010–2014 (3).

In previous studies, the clinicopathological characteristics of lung cancer have changed in many countries during the past 20 years. An increasing percentage of asymptomatic patients, females and nonsmokers among lung cancer patients has been demonstrated. Additionally, studies have shown increasing trends of adenocarcinoma along with decreasing trends of squamous cell carcinoma (4, 5). Many studies have demonstrated that screening high-risk populations with low-dose computed tomography (LDCT) can improve the early detection rate and reduce the mortality of lung cancer (6). Moreover, the surgical treatments for lung cancer have also changed during the past decades. During the last decade, the number of procedures with the open thoracotomy approach has decreased, but minimally invasive approaches, including video-assisted thoracic surgery (VATS) and robot-assisted thoracic surgery (RATS), have become increasingly dominant (7).

Although the above trends of the clinicopathological characteristics and surgical treatments of lung cancer have been sporadically presented in multiple studies, those in China have never been comprehensively described. In the current study, we collected and analyzed the medical data of operable primary lung cancer patients in the Department of Thoracic Surgery of Sun Yat-sen University Cancer Center (SYSUCC) from 2011 to 2020. We aim to explore the developing trends of the clinicopathological characteristics, surgical treatments and survival outcomes of operable lung cancer patients within the past 10 years and to provide valuable insights for the future management of the disease.

## Methods

We conducted a retrospective analysis of operable lung cancer patients who underwent surgical treatment in the Department of Thoracic Surgery of SYSUCC from January 1st 2011 to December 31st 2020. Our study was approved by the Institutional Review Board of SYSUCC (NO. B2023-167-01). And Individual consent for this retrospective analysis was waived.

### Patient selection and data extraction

Medical records of primary operable lung cancer patients treated from January 2011 to December 2020 were screened within database of SYSUCC. To ensure the accuracy of the medical data, we further supplied and calibrated some of the missing data by reviewing the original medical records. The 8th edition American Joint Committee on Cancer (8th AJCC) TNM stage was extracted, and the previous edition TNM staging was systemically converted into the 8th AJCC TNM stage in our study. The specific inclusion criteria were as follows: (1) patients diagnosed with primary operable lung cancer; (2) patients confirmed with positive histopathological assessment. The exclusion criteria were as follows: (1) patients with unknown medical records and (2) patients with second primary lung cancer or other primary malignant tumors during the follow-up period. In addition, the follow-up deadline was set at December 31st, 2021, and the survival time was defined as the interval between surgery and death with patients without event being censored at the last follow-up. We further collected patients with a follow-up time of at least 30 days for survival analysis (Supplementary Figure 1).

### Statistical analysis

In our study, the clinicopathological characteristics, including age, clinical manifestation, gender, smoking history, tumor size, histology and stage, were obtained from medical records. Additionally, we collected information on the surgical treatments, including surgical approach, extent of resection, lymph node dissection, postoperative length of stay and postoperative mortality from the surgical records and medical records. Moreover, overall survival (OS) was set as the primary survival outcome, and 1-year, 3-year, 5-year, and 10-year OS were calculated by using the Kaplan–Meier method. Quantitative variables were reported using the mean with the standard error, and the categorical variables were described with percentages. One-way ANOVA was used to compare the means of quantitative variables between different groups. The chi-square test was used to compare the percentage of categorical variables between different groups. A two-tailed *p*-value <0.05 was considered statistically significant.

## Results

### Trends in the clinicopathological characteristics

**Figure 1:**
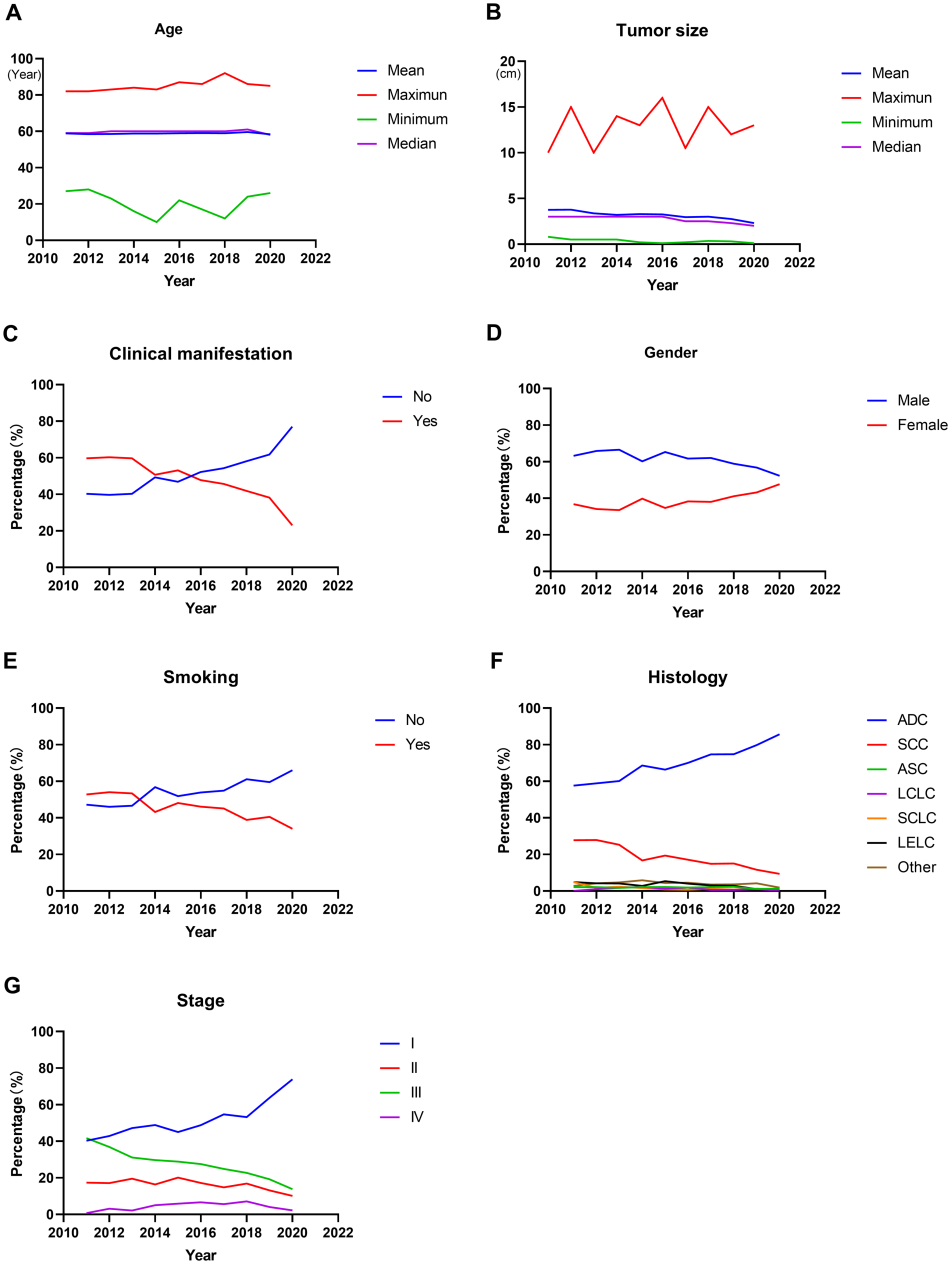
Trends in clinicopathological characteristics in operable lung cancer patients from 2011 to 2020. **(A)** Patients’ age at diagnosis. **(B)** Tumor size. **(C)** Percentage of patients with or without clinical manifestation. **(D)** Percentage of male or female patients. **(E)** Percentage of patients with or without smoking history. **(F)** Percentage of patients with different histological types. **(G)** Percentage of patients with different stages. ADC, adenocarcinoma; SCC, squamous cell carcinoma; ASC, adenosquamous carcinoma; LCLC, large cell lung cancer; SCLC, small cell lung cancer; LELC, lymphoepithelioma-like carcinoma.

A total of 7,800 operable lung cancer patients were enrolled in our study, and the number of patients with different clinicopathological characteristics and surgical treatments was shown in Supplementary Table 1. Among these patients, the average age at diagnosis of the patients was 58.854 ± 10.185 years. There was no significant change in the average age at diagnosis during the last 10 years (*p* = 0.336, Figure 1A; Table 1). However, the average tumor diameter decreased significantly from 3.776 ± 2.062 cm in 2011 to 2.300 ± 1.512 cm in 2020 over the last decade (*p* < 0.001, Figure 1B; Table 1). The ratio of the asymptomatic patients accounted for 56.6%, and increased significantly from 40.3% in 2011 to 77.0% in 2020 over the decade (*p* < 0.001, Figure 1C; Table 1). Male patients accounted for 59.8%, but the percentage of female patients increased significantly from 36.8% in 2011 to 47.7% in 2020 (*p* < 0.001, Figure 1D; Table 1). Overall, the proportion of patients with a smoking history accounted for 42.9%, which decreased significantly from 52.8% in 2011 to 34.0% in 2020 over the years (*p* < 0.001, Figure 1E; Table 1).

**Table 1:** Clinicopathological characteristics of operable lung cancer patients from 2011 to 2020.

Features	2011	2012	2013	2014	2015	2016	2017	2018	2019	2020	Total	*p*-value
Age												0.363
Mean ± SE (year)	58.903 ± 10.714	58.467 ± 9.383	58.513 ± 10.287	58.806 ± 10.698	58.773 ± 10.329	58.929 ± 9.961	59.077 ± 10.084	58.936 ± 10.006	59.623 ± 10.047	58.338 ± 10.387	58.854 ± 10.185	
Clinical manifestation												<0.001
No	40.3%	39.7%	40.3%	49.3%	46.9%	52.2%	54.3%	58.1%	61.8%	77.0%	56.6%	
Yes	59.7%	60.3%	59.7%	50.7%	53.1%	47.8%	45.7%	41.9%	38.2%	23.0%	43.4%	
Gender												<0.001
Male	63.2%	65.9%	66.5%	60.2%	65.3%	61.7%	62.0%	58.9%	56.8%	52.3%	59.8%	
Female	36.8%	34.1%	33.5%	39.8%	34.7%	38.3%	38.0%	41.1%	43.2%	47.7%	40.2%	
Smoking												<0.001
No	47.2%	46.0%	46.6%	56.8%	51.9%	53.9%	54.9%	61.1%	59.5%	66.0%	57.1%	
Yes	52.8%	54.0%	53.4%	43.2%	48.1%	46.1%	45.1%	38.9%	40.5%	34.0%	42.9%	
Tumor size												<0.001
Mean ± SE (cm)	3.766 ± 2.062	3.777 ± 2.267	3.377 ± 1.783	3.212 ± 1.889	3.284 ± 1.801	3.256 ± 2.052	2.954 ± 1.813	3.014 ± 1.866	2.750 ± 1.851	2.300 ± 1.512	2.990 ± 1.875	
Histology												<0.001
ADC	57.6%	58.9%	60.1%	68.6%	66.4%	70.0%	74.7%	74.8%	79.8%	85.7%	73.5%	<0.001
SCC	27.8%	27.9%	25.3%	16.7%	19.4%	17.1%	14.9%	15.0%	11.6%	9.4%	15.8%	<0.001
ASC	2.1%	2.1%	1.7%	2.1%	2.2%	1.9%	2.1%	2.3%	1.1%	1.3%	1.8%	0.515
LCLC	0%	1.0%	1.7%	2.3%	1.2%	1.9%	0.6%	0.6%	1.0%	0.3%	1.0%	<0.001
SCLC	4.9%	1.7%	2.4%	1.6%	0.9%	0.7%	1.2%	0.8%	1.3%	0.9%	1.2%	0.001
LELC	4.9%	4.2%	4.1%	2.8%	5.4%	4.0%	3.0%	2.9%	1.0%	0.6%	2.8%	<0.001
Other	2.7%	4.2%	4.7%	5.9%	4.5%	4.4%	3.5%	3.6%	4.2%	1.8%	3.9%	0.002
Stage												<0.001
I	40.3%	42.9%	47.2%	48.9%	45.0%	48.8%	54.7%	53.2%	63.8%	73.9%	55.5%	<0.001
II	17.4%	17.1%	19.5%	16.4%	20.1%	17.2%	14.8%	16.9%	13.1%	10.1%	15.5%	<0.001
III	41.7%	36.9%	31.1%	29.7%	28.9%	27.5%	24.9%	22.7%	19.2%	13.8%	24.2%	<0.001
IV	0.7%	3.1%	2.1%	5.0%	5.9%	6.6%	5.6%	7.1%	4.0%	2.2%	4.8%	<0.001

SE, standard error; ADC, adenocarcinoma; SCC, squamous cell carcinoma; ASC, adenosquamous carcinoma; LCLC, large cell lung cancer; SCLC, small cell lung cancer; LELC, lymphoepithelioma-like carcinoma.

Among these patients, the majority were adenocarcinoma (ADC) and squamous cell carcinoma (SCC), accounting for 73.5 and 15.8%, respectively. Furthermore, the percentage of ADC increased significantly from 57.6% in 2011 to 85.7% in 2020 (*p* < 0.001, Figure 1F; Table 1), with a significant decrease in SCC (from 27.8% in 2011 to 9.4% in 2020, *p* < 0.001, Figure 1F; Table 1). And the stage I, II, III and IV patients accounted for 55.5, 15.5, 24.2, and 4.8%, respectively. Moreover, the ratio of stage I increased significantly from 40.3% in 2011 to 73.9% in 2020 (*p* < 0.001, Figure 1G; Table 1), accompanied by significant decreases in the proportion of stage II and stage III (both *p* < 0.001, Figure 1G; Table 1). In addition, there was also a significant change in the proportion of stage IV patients, which increased from 0.7% in 2011 to 7.1% in 2018 and then decreased to 2.2% in 2020 (*p* < 0.001, Figure 1G; Table 1).

### Trends in surgical treatments

**Figure 2:**
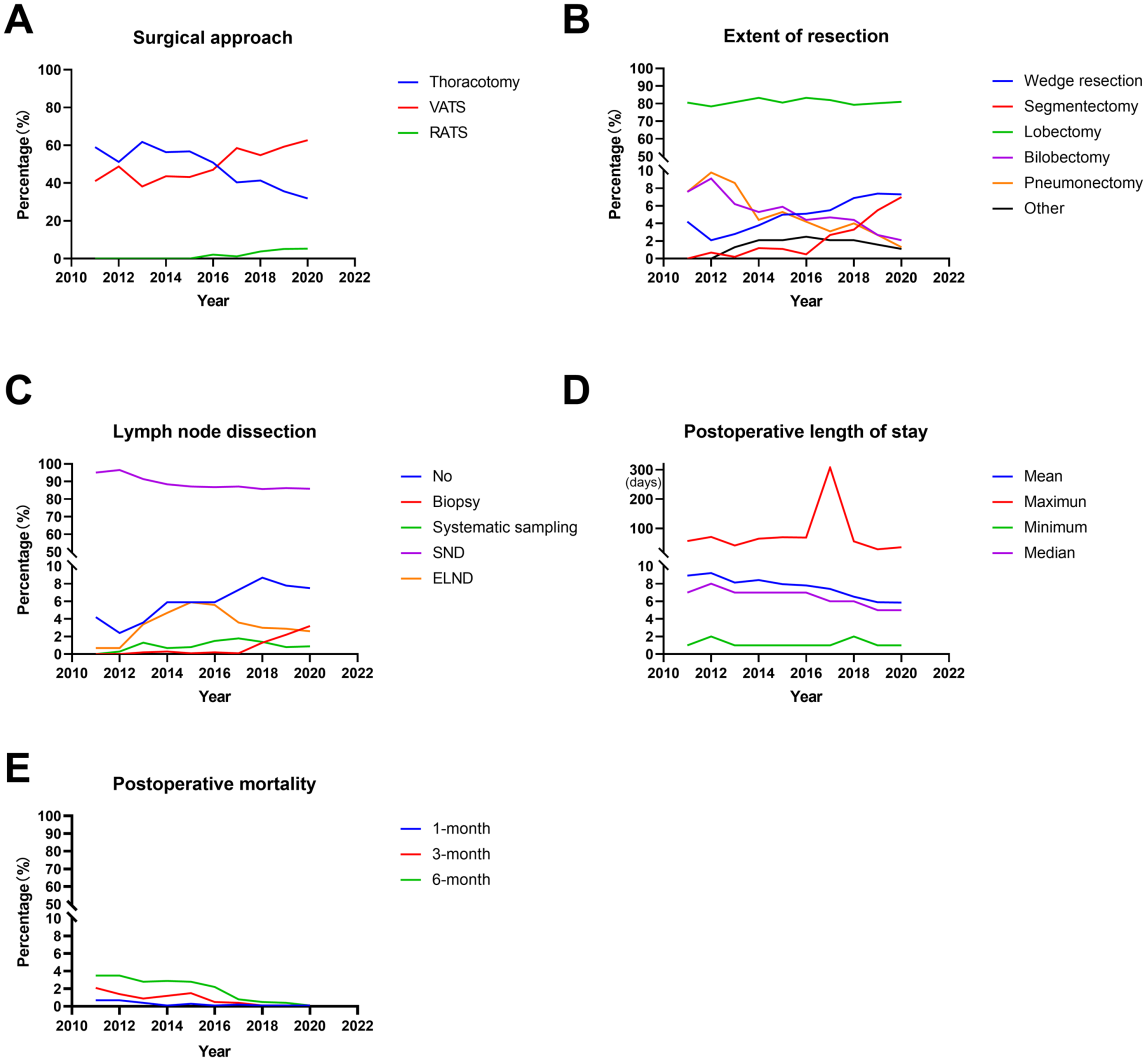
Trends in surgical treatments in operable lung cancer patients from 2011 to 2020. **(A)** Percentage of different surgical methods. **(B)** Postoperative length of stay. **(C)** Percentage of different surgical procedure. **(D)** Percentage of different lymph node dissection. **(E)** Postoperative mortality. VATS, video-assisted thoracic surgery; RATS, robot-assisted thoracic surgery; SND, systematic nodal dissection; ELND, extended lymph node dissection.

During the 10 years, the majority of the surgical approaches were minimally invasive surgeries, including VATS and RATS, which accounted for 52.7 and 2.5% of all surgeries, respectively. Moreover, the proportion of VATS increased significantly from 41.0% in 2011 to 62.7% in 2020 (*p* < 0.001, Figure 2A; Table 2) and that of thoracotomy decreased significantly from 59.0% in 2011 to 31.9% in 2015 (*p* < 0.001, Figure 2A; Table 2). In addition, the RATS was initiated in 2016 and increased from 2.1% in 2016 to 5.4% in 2020 (*p* < 0.001, Figure 2A; Table 2).

**Table 2:** Surgical treatment of operable lung cancer patients from 2011 to 2020.

Features	2011	2012	2013	2014	2015	2016	2017	2018	2019	2020	Total	*p*-value
Surgical approach												<0.001
Thoracotomy	59.0%	51.2%	61.8%	56.4%	56.8%	50.9%	40.4%	41.4%	35.6%	31.9%	44.8%	<0.001
VATS	41.0%	48.8%	38.2%	43.6%	43.2%	47.0%	58.5%	54.8%	59.3%	62.7%	52.7%	<0.001
RATS	0%	0%	0%	0%	0%	2.1%	1.1%	3.8%	5.1%	5.4%	2.5%	<0.001
Extent of resection												<0.001
Wedge resection	4.2%	2.1%	2.8%	3.8%	5.0%	5.1%	5.5%	6.9%	7.4%	7.3%	5.7%	<0.001
Segmentectomy	0%	0.7%	0.2%	1.2%	1.1%	0.5%	2.7%	3.3%	5.5%	7.0%	3.0%	<0.001
Lobectomy	80.6%	78.4%	80.9%	83.3%	80.6%	83.3%	82.0%	79.3%	80.2%	81.0%	81.1%	0.357
Bilobectomy	7.6%	9.1%	6.2%	5.3%	5.9%	4.4%	4.7%	4.4%	2.7%	2.1%	4.4%	<0.001
Pneumonectomy	7.6%	9.8%	8.6%	4.4%	5.3%	4.2%	3.1%	4.0%	2.7%	1.3%	4.0%	<0.001
Other	0%	0%	1.3%	2.0%	2.1%	2.5%	2.0%	2.1%	1.5%	1.3%	1.8%	0.047
Lymph node dissection												<0.001
No	4.2%	2.4%	3.6%	5.9%	5.9%	5.9%	7.3%	8.7%	7.8%	7.5%	6.7%	<0.001
Biopsy	0%	0%	0.2%	0.3%	0.1%	0.2%	0.1%	1.3%	2.2%	3.2%	1.1%	<0.001
Systematic sampling	0%	0.3%	1.3%	0.7%	0.8%	1.5%	1.8%	1.4%	0.8%	0.9%	1.1%	0.183
SND	95.1%	96.5%	91.4%	88.4%	87.2%	86.8%	87.2%	85.7%	86.3%	85.9%	87.4%	<0.001
ELND	0.7%	0.8%	3.5%	4.7%	6.0%	5.6%	3.6%	3.0%	2.9%	2.5%	3.7%	<0.001
Postoperative length of stay												<0.001
Mean ± SE (days)	8.924 ± 6.330	9.213 ± 6.423	8.137 ± 4.346	8.427 ± 5.064	7.962 ± 5.150	7.816 ± 5.556	7.412 ± 10.659	6.538 ± 3.653	5.904 ± 2.881	5.862 ± 3.414	7.170 ± 5.945	
1-month postoperative mortality	0.7%	0.7%	0.4%	0.1%	0.3%	0.1%	0.2%	0.1%	0.1%	0.1%	0.2%	0.450
3-month postoperative mortality	2.1%	1.4%	0.9%	1.2%	1.5%	0.5%	0.4%	0.1%	0.1%	0.1%	0.6%	<0.001
6-month postoperative mortality	3.5%	3.5%	2.8%	2.9%	2.8%	2.2%	0.8%	0.5%	0.4%	0.1%	1.4%	<0.001

SE, standard error; VATS, video-assisted thoracic surgery; RATS, robot-assisted thoracic surgery; SND, systematic nodal dissection; ELND, extended lymph node dissection.

Regarding the extent of resection, the majority of the patients underwent lobectomy, accounting for 81.1%. Wedge resection, segmentectomy, bilobectomy and pneumonectomy accounted for 5.7, 3.0, 4.4, and 4.0%, respectively. Moreover, lobectomies remained stable for 10 years, accounting for ~80% (*p* = 0.357, Figure 2B; Table 2). In addition, there were significant increases in the proportion of wedge resection and segmentectomy (both *p* < 0.001, Figure 2B; Table 2). However, the ratio of bilobectomy and pneumonectomy decreased significantly over 10 years (both *p* < 0.001, Figure 2B; Table 2).

In terms of lymph node dissection, the majority of the patients underwent systematic nodal dissection (SND), accounting for 87.4%. The percentages of the patients with no lymph node dissection, biopsy, systematic sampling and extended lymph node dissection (ELND) were 6.7, 1.1, 1.1, and 3.7%, respectively. Among these patients, the percentage of systematic sampling remained stable over the decade (*p* = 0.183, Figure 2C; Table 2). However, the ratio of SND decreased significantly from 95.1% in 2011 to 85.9% in 2020 (*p* < 0.001, Figure 2C; Table 2), with the significant increases in no lymph node dissection and biopsy (both *p* < 0.001, Figure 2C; Table 2). In addition, a two-stage development trend was observed in ELND: the proportion increased from 0.7% in 2011 to 6.0% in 2015 and then decreased to 2.5% in 2020 (*p* < 0.001, Figure 2C; Table 2). During the decade, the average postoperative length of stay was 7.170 ± 5.945 days. Furthermore, the average postoperative length of stay decreased significantly from 8.924 ± 6.330 days in 2011 to 5.862 ± 3.414 days in 2020 (*p* < 0.001, Figure 2D; Table 2). In addition, the 1-month postoperative mortality decreased from 0.7% in 2011 to 0.1% (*p* = 0.450, Figure 2E; Table 2), with the significant decreases in the 3-month and 6-month postoperative mortality (from 2.1 and 3.5% in 2011 to 0.1 and 0.1%, respectively, both *p* < 0.001, Figure 2E; Table 2).

### Trends in survival outcomes

**Figure 3:**
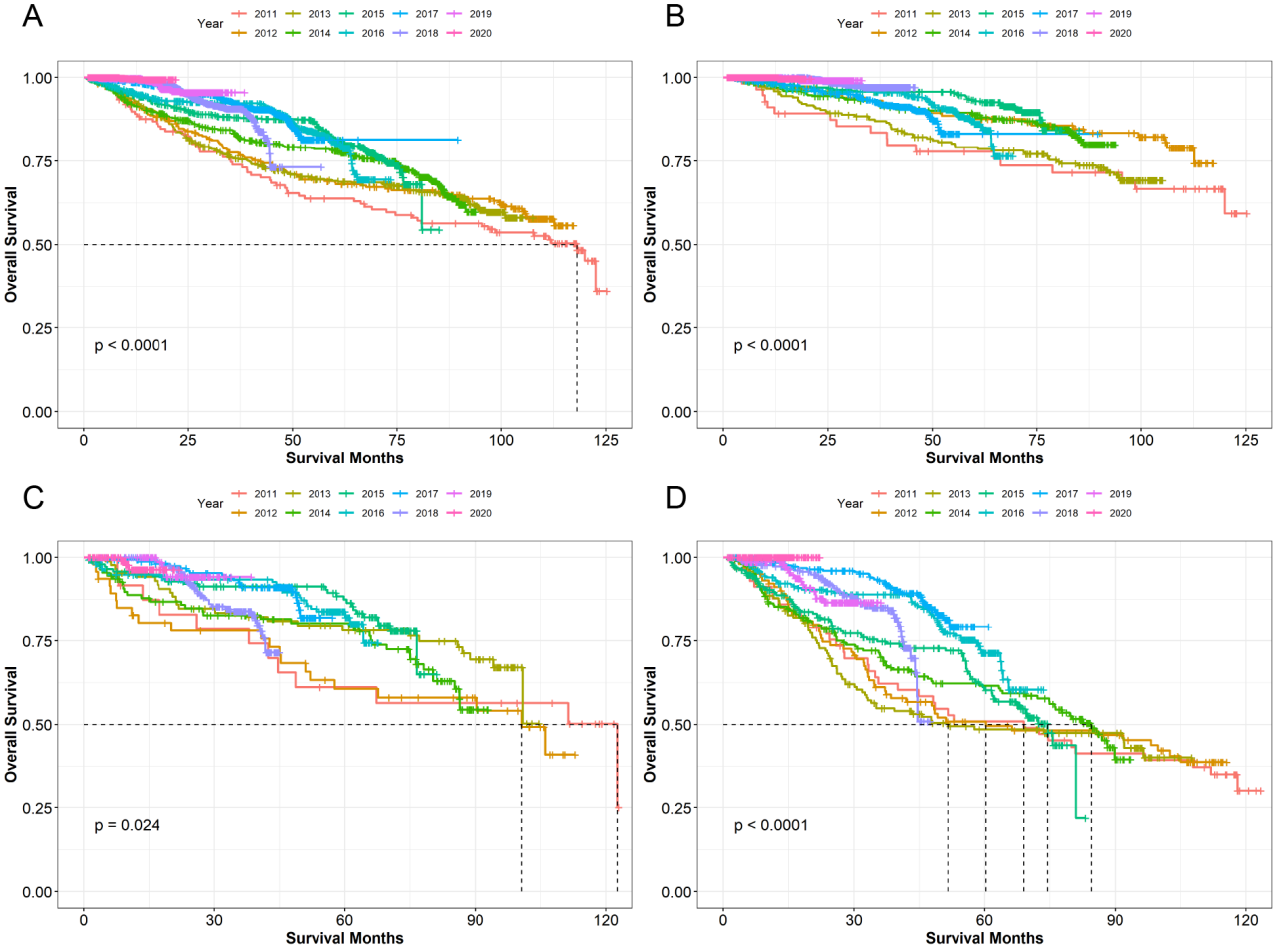
Kaplan–Meier survival curves of the operable lung cancer patients treated in different years among different stages. **(A)** All selected patients. **(B)** Patients diagnosed with stage I. **(C)** Patients diagnosed with stage II. **(D)** Patients diagnosed with stage III.

In our study, 7575 patients with a follow-up of at least 30 days were included for the survival analysis. Among these patients, the shortest and longest follow-ups were 30 days and 3759 days, respectively, with a median follow-up of 1065 days. Among these patients, the 1-year, 3-year, 5-year and 10-year OS rates were 96.8, 87.7, 76.9, and 48.4%, respectively. Moreover, there were significant increases in 1-year, 3-year and 5-year OS (from 89.8, 73.9, and 63.8% in 2011 to 99.6% in 2020, 90.7% in 2018 and 80.8% in 2016, respectively, all *p* < 0.001, Supplementary Table 2; Figure 3A) over the years.

Since the majority of the operable lung cancer patients were diagnosed with stage I-III disease, we further analyzed the OS of the stage I-III patients. For stage I, similarly significant increases were observed in 1-year, 3-year and 5-year OS (from 91.0, 83.5, and 77.8% in 2011 to 100% in 2020, 97.1% in 2018 and 87.4% in 2016, respectively, all *p* ≤ 0.001, Table 3; Figure 3B). In terms of stage II, 1-year and 3-year OS increased significantly from 91.7 and 78.6% in 2011 to 96.3% in 2020 and 83.8% in 2018, respectively (*p* < 0.001 and *p* = 0.028, respectively, Table 3; Figure 3C). In addition, 5-year OS increased from 61.1% in 2011 to 83.8% in 2016 (*p* = 0.078, Table 3; Figure 3C). For the stage III, 1-year and 3-year OS increased significantly from 87.7 and 62.2% in 2011 to 100% in 2020 and 85.0% in 2018, respectively (*p* < 0.001, Table 3; Figure 3D). And 5-year OS also increased from 50.9% in 2011 to 71.4% in 2016 (*p* = 0.090, Table 3; Figure 3D).

**Table 3:** Overall survival of operable lung cancer patients with different stage from 2011 to 2020.

Year	Overall survival of stage I				Overall survival of stage II				Overall survival of stage III			
	1-year	3-year	5-year	10-year	1-year	3-year	5-year	10-year	1-year	3-year	5-year	10-year
2011	91.0%	83.5%	77.8%	59.2%	91.7%	78.6%	61.1%	50.1%	87.7%	62.2%	50.9%	30.1%
2012	96.6%	93.0%	88.5%		82.7%	78.1%	68.3%		91.2%	61.1%	50.8%	
2013	96.8%	87.4%	79.1%		94.2%	83.4%	78.2%		87.8%	54.8%	48.5%	
2014	97.5%	92.8%	89.1%		88.7%	82.7%	80.3%		85.2%	70.2%	61.5%	
2015	98.2%	95.7%	92.9%		95.1%	91.3%	87.4%		89.2%	74.8%	60.3%	
2016	98.1%	95.7%	87.4%		94.8%	93.4%	83.8%		93.1%	88.9%	71.4%	
2017	98.5%	92.9%			99.4%	91.8%			98.5%	93.0%		
2018	100%	97.1%			100%	83.8%			97.3%	85.0%		
2019	100%				100%				98.7%			
2020	100%				96.3%				100%			
Total	98.8%	94.0%	87.6%		95.9%	87.6%	79.9%		93.5%	79.9%	59.9%	
*p*-value	<0.001	<0.001	0.001		<0.001	0.028	0.078		<0.001	<0.001	0.090	

## Discussion

The results of our study were based on data collected in the largest cancer center in South China during the decade. With the large sample size, the results could reflect at some extent the overall Chinese operable lung cancer population over the decade. Furthermore, as far as we know, it is the first study to objectively reflect the last 10-year trends of clinicopathological characteristics, surgical treatments and survival outcomes of operable lung cancer patients in a monocenter of China.

In our study, there was no significant change in the average age at diagnosis during the decade. And the study from the National Cancer Center consistently demonstrated that the adjusted mean age of lung cancer patients at diagnosis remained stable during 2000–2014 in China (8). These findings indicated that the average age of operable lung cancer onset in China was relatively stable. In addition, the National Lung Screening Trial (NLST) demonstrated the significance of LDCT for the early screening of lung cancer, and the NCCN guidelines further recommended LDCT as an effective screening method for lung cancer (9, 10). Owing to the increasing application of LDCT, more asymptomatic lung cancer patients were detected and the ratio of them increased over the decade. Comparing the Global Cancer Statistics from 2012 with those from 2020, the incidence rate of lung cancer in males was lower in 2020 than in 2012, but it was reversed for women. However, there were still more new lung cancer cases in males than females in 2020. The above results explained the majority of male operable lung cancer patients and the increasing percentage of female patients (1, 11). In previous studies, smoking had been proven to be a high risk factor for lung cancer, causing ~85% of lung cancer cases, and secondhand smoke exposure had a great impact on nonsmokers (12). Recently, the prevalence of smoking among both males and females has decreased steadily in China (13). Therefore, the decreasing percentage of male patients may be related to the decrease in smoking prevalence, and the increasing ratio of female patients may result from secondhand smoke exposure, occupational exposure and air pollution (14). At present, lung cancer screening program is still implemented according to the NLST eligibility criteria, focusing on the smokers and the elderly. However, our research suggested that non-smokers and female need more attention in China. The above observations indicated that more appropriate eligible criteria need to be established for people outside the United States, which was consistently demonstrated in previous study from Taiwan (15). Our results revealed that the tumor size of operable lung cancer patients decreased over 10 years, with increasing ratio of early stage patients. The increase in the early-stage detection rate among the operable lung cancer patients was attributed to the emphasis on lung cancer screening (16). Moreover, the distinct stage shift was also observed in other previous research, consistently demonstrating the close correlation between early detection and implementation of LDCT in lung cancer patients (17). However, although the percentage of stage IV patients remained at a very low level, its increasing trend followed by a decreasing trend was also noteworthy in our study. An earlier study showed the survival benefits of surgery among lung cancer patients with oligometastases, which explained the increasing trend (18). However, a recent study further demonstrated that surgery should be considered much less often for stage IV patients with mediastinal nodal disease or more locally advanced tumors, which also served as a basis for the decreasing trend in stage IV patients in our study (19). Moreover, our study revealed an increasing proportion of adenocarcinoma and a decreasing proportion of squamous cell carcinoma, which was consistent with the previous study (20). Smoking has been proven to be more associated with squamous cell lung cancer, while adenocarcinoma was more likely to be seen in nonsmokers (21). Therefore, the decreasing prevalence of smoking may be attributed to the changes in the histological types.

At present, the use of VATS has become the standard approach for the surgical resection of early-stage lung cancer (22). Our study consistently demonstrated that VATS and RATS as minimally invasive surgery accounted for the majority of the surgeries performed in operable lung cancer patients. Although RATS accounted for a small proportion and had a high surgical cost, its confirmed advantage as VATS and rapid postoperative recovery also indicated its promising prospects (23, 24). In addition, a lower incidence of complications was proven in minimally invasive surgery than thoracotomy (25). Owing to the increase in minimally invasive surgery, a decreasing length of postoperative hospital stay was also observed in our study. A meta-analysis demonstrated that sublobectomy produced similar survival to lobectomy for stage IA patients with tumors ≤2 cm (26). With the increasing ratio of early-stage patients, the increasing trend of sublobectomy was also observed in our study. In the previous study, the improvement on procedure and extent of lung resection was consistently proved to be beneficial from more early-stage patients detected by LDCT (27). Although sublobectomy has the advantages of low complication, lobectomy remains the safest and oncologically correct method in lung cancer patients (28). Therefore, lobectomy remained the majority of the surgical procedures. In our study, the different methods of lymph node dissection changed significantly, but SND still accounted for the majority. And the American College of Surgery Oncology Group (ACSOG) Z0030 trial demonstrated that systematic sampling had the same survival as SND in early-stage lung cancer patients, but SND was still recommended as the standard method (29). A recent study showed that video-assisted mediastinoscopic lymphadenectomy (VAMLA) as ELND should be the preferred technique with superior sensitivity and negative predictive value in detecting positive mediastinal lymph nodes and with an increased risk of complications (30). This further explained that the proportion of ELND increased first and then decreased in our study, which also reflected its active exploration and cautious application in our cancer center. Previous studies have proven that decreasing postoperative mortality can be caused by increasing early-stage lung cancer, which explains the decreasing mortality and improved surgical safety in our study (31).

Our study showed that the 1-year, 3-year, and 5-year OS increased in operable lung cancer patients. Owing to the important role of surgical treatment in stage I-IIIA lung cancer patients, we further analyzed the trends of OS in the stage I–III patients in our research. The results suggested that 1-year, 3-year, and 5-year OS increased in all stages, including stages I–III. In a previous study, postoperative adjuvant treatments, including chemotherapy, radiotherapy, immunotherapy and targeted therapy, were proven to be effective in operable lung cancer patients but still had limitations (32). Therefore, this also explained why there was no significant increase in 5-year OS of stages II and III patients in our study. Moreover, the 5-year OS of every stage in our study was superior to that of an international multicenter study, whose 5-year OS of stage I–III were 80.3, 60.2, and 40.6%, respectively (33). In addition, the 5-year OS superiority of our study was also proven again by comparing the survival results from Japan (20). The curative effect of operable lung cancer patients in our cancer center has reached an internationally advanced level.

However, there were also some limitations to our study. First, our study was a mono-center, retrospective observational study, so the results from our research should be further validated in other medical centers. Second, as the follow-up of the patients from 2017 to 2020 was <5 years, the 5-year OS of these patients was not analyzed. Thus, the objectivity and accuracy of the long-term survival analysis may be affected. Additionally, we analyzed the 10-year trends of the patients from 2011 to 2020, and whether there were similar trends in the patients in earlier years should be further explored.

## Conclusion

In our study, we investigated the past 10-year trends of the clinicopathological characteristics, surgical treatments and survival outcomes of 7,800 operable lung cancer patients in SYSUCC, which could at some extent reflect the trends of Chinese operable lung cancer patients. Our results showed that there was an increasing percentage of asymptomatic, female, nonsmoking, and early-stage patients with adenocarcinoma. With the development of minimally invasive surgery, surgical procedures and lymph node dissection, postoperative recovery has become faster and safer, and the survival outcome has improved, which has reached the international advanced level. Moreover, this is the first comprehensive review of the trends in operable lung cancer patients form a monocenter of China during the past decade, which can deliver critical insights for future studies and the standard of care for operable lung cancer patients.

## Data availability statement

The raw data supporting the conclusions of this article will be made available by the authors, without undue reservation.

## Ethics statement

Our study was approved by the Institutional Review Board of Sun Yat-sen University Cancer Center (NO. B2023-167-01). Individual consent for this retrospective analysis was waived.

## Author contributions

LZ, DZ, and XH: conception and design. LC and LZ: administrative support. RZ, YW, and XZ: provision of study materials or patients. GW and GG: collection and assembly of data. DZ and XH: data analysis and interpretation. DZ, XH, RZ, ZH, YW, XZ, GW, GG, LC, and LZ: manuscript writing. All authors contributed to the article and approved the submitted version.

## Conflict of interest

The authors declare that the research was conducted in the absence of any commercial or financial relationships that could be construed as a potential conflict of interest.

## Publisher’s note

All claims expressed in this article are solely those of the authors and do not necessarily represent those of their affiliated organizations, or those of the publisher, the editors and the reviewers. Any product that may be evaluated in this article, or claim that may be made by its manufacturer, is not guaranteed or endorsed by the publisher.

## Figures and Tables

**Table:** 

SYSUCC	Sun Yat-sen University Cancer Center
LDCT	low-dose computed tomography
OS	overall survival
ICD-O	International Classification of Diseases for Oncology
ADC	adenocarcinoma
SCC	squamous cell carcinoma
ASC	adenosquamous carcinoma
LCLC	large cell lung cancer
SCLC	small cell lung cancer
LELC	lymphoepithelioma-like carcinoma
AJCC	American Joint Committee on Cancer
VATS	video-assisted thoracic surgery
RATS	robot-assisted thoracic surgery
SND	systematic nodal dissection
ELND	extended lymph node dissection
VAMLA	video-assisted mediastinoscopic lymphadenectomy
NLST	National Lung Screening Trial
ACSOG	American College of Surgery Oncology Group
