# Supplementary material for: Ten-year trends of the clinicopathological characteristics, surgical treatments and survival outcomes of operable lung cancer patients in monocenter: a retrospective cohort study
Source: Front Med (Lausanne). 2023 Apr 26;10:1133344. doi: 10.3389/fmed.2023.1133344 (PMC10169745; doi:10.3389/fmed.2023.1133344)
Supplement: Supplementary file 1 [file Table_1.DOCX]

**Supplementary Table 1.** **The number of patients among different clinicopathological characteristics and surgical treatments**

| Features | Patients |
| --- | --- |
| Total | 7800 |
| Clinical manifestation |  |
| No | 4416 |
| Yes | 3384 |
| Gender |  |
| Male | 4667 |
| Female | 3133 |
| Smoking |  |
| No | 4454 |
| Yes | 3346 |
| Histology |  |
| ADC | 5733 |
| SCC | 1233 |
| ASC | 144 |
| LCLC | 79 |
| SCLC | 93 |
| LELC | 222 |
| Other | 296 |
| Stage |  |
| I | 4326 |
| II | 1208 |
| III | 1891 |
| IV | 375 |
| Surgical approach |  |
| Thoracotomy | 3496 |
| VATS | 4110 |
| RATS | 194 |
| Extent of resection |  |
| Wedge resection | 444 |
| Segmentectomy | 236 |
| Lobectomy | 6327 |
| Bilobectomy | 345 |
| Pneumonectomy | 312 |
| Other | 136 |
| Lymph node dissection |  |
| No | 525 |
| Biopsy | 84 |
| Systematic sampling | 88 |
| SND | 6819 |
| ELND | 284 |

ADC: adenocarcinoma; SCC: squamous cell carcinoma; ASC: adenosquamous carcinoma; LCLC: large cell lung cancer; SCLC: small cell lung cancer; LELC: lymphoepithelioma-like carcinoma; VATS: video-assisted thoracic surgery; RATS: robot-assisted thoracic surgery; SND: systematic nodal dissection; ELND: extended lymph node dissection
