# Supplementary material for: Ten-year trends of the clinicopathological characteristics, surgical treatments and survival outcomes of operable lung cancer patients in monocenter: a retrospective cohort study
Source: Front Med (Lausanne). 2023 Apr 26;10:1133344. doi: 10.3389/fmed.2023.1133344 (PMC10169745; doi:10.3389/fmed.2023.1133344)
Supplement: Supplementary file 2 [file Table_2.DOCX]

**Supplementary Table 2. Overall survival of operable lung cancer patients from 2011 to 2020**

| Year | Overall survival | | | |
| --- | --- | --- | --- | --- |
|  | 1-year | 3-year | 5-year | 10-year |
| 2011 | 89.8% | 73.9% | 63.8% | 48.4% |
| 2012 | 92.3% | 77.6% | 69.0% |  |
| 2013 | 93.4% | 76.0% | 69.0% |  |
| 2014 | 91.7% | 83.2% | 78.3% |  |
| 2015 | 94.7% | 88.0% | 81.4% |  |
| 2016 | 95.4% | 92.4% | 80.8% |  |
| 2017 | 98.6% | 92.3% |  |  |
| 2018 | 99.2% | 90.7% |  |  |
| 2019 | 99.7% |  |  |  |
| 2020 | 99.6% |  |  |  |
| Total | 96.8% | 87.7% | 76.9% |  |
| *P* | ＜.001 | ＜.001 | ＜.001 | ＜.001 |
